# Legal issues associated with antimicrobial drug resistance.

**DOI:** 10.3201/eid0402.980204

**Published:** 1998

**Authors:** D P Fidler

**Affiliations:** Indiana University School of Law, Bloomington, USA. davidfidler@law.indiana.edu

## Abstract

An effective public health strategy against the development of antimicrobial drug resistance needs to be informed by legal as well as scientific analysis. This article describes some legal issues arising from current efforts against antimicrobial resistance and underscores the interdependence between law and public health in these efforts.


### Legal Issues Associated with Antimicrobial Drug Resistance

**David P. Fidler**
Indiana University School of Law, Bloomington, Indiana, USA

An effective public health strategy against the development of antimicrobial drug resistance needs to be informed by legal as well as scientific analysis. This article describes some legal issues arising from current efforts against antimicrobial resistance and underscores the interdependence between law and public health in these efforts.

The development of antimicrobial resistance in many pathogenic microbes poses one of the most serious problems in the control of infectious diseases (1-3). Antimicrobial resistance results from the ability of microbes to adapt to anthropogenic pressures; therefore, it is not a passing trend but likely a permanent feature in the fight against infectious diseases (4). This article highlights the legal issues involved in addressing the problem of antimicrobial resistance.

### Law and Global Public Health

Globalization interferes with infectious disease control at the national level (1,5-7). While microbes move freely around the world, unhindered by borders, human responses to infectious diseases are conditioned by jurisdictional boundaries. Therefore, public health responses to infectious diseases must constantly navigate the mazes created by international and national law. Although national law now dominates legal approaches to infectious diseases, the global nature of the emerging infectious disease problem points towards a larger future role for international law.

Especially in federal systems, countries often divide authority for public health among various levels of government. In the United States, for example, states have primary power for public health because the Constitution did not grant the federal government explicit public health powers (8). While the federal government has authority to act in the public health context (9), its statutes and regulations derive from other federal powers. Most U.S. public health law is at the state level. The emerging infectious disease threat points to a larger role not only for international but also for federal law. Federal agencies create networks that allow the state and national governments to cooperate on issues such as antimicrobial resistance, but public health law remains primarily a state domain; therefore, state laws on public health may need to be reevaluated in the context of emerging infectious disease control (10).

Address for correspondence: David P. Fidler, Indiana University School of Law, 211 South Indiana Avenue, Bloomington, IN 47405-1001, USA; fax: 812-855-0555; e-mail: davidfidler@law.indiana.edu.

### Public Health Strategies to Address Antimicrobial Resistance

The dominant public health strategy against antimicrobial resistance contains improved surveillance of resistant pathogens, as well as rational use and increased research and development of new antimicrobial drugs (1). These elements fit within the larger strategy to address emerging infectious diseases, which stresses surveillance, applied research, prevention and control, and infrastructure development (1).

### Surveillance

Surveillance is critical to the control and prevention of infectious diseases (1). Since no national or global surveillance system exists for monitoring antibiotic resistance, improved surveillance is a top priority (11). Surveillance is also useful in addressing the threat of biological weapons and protecting the community from highly contagious infectious diseases. Law is critical to surveillance not only because reporting information must be a legal duty, but also because law is needed to deal with the tensions sometimes arising between individual privacy rights and the community's interest in being protected from infectious diseases.

### Reporting

International and domestic surveillance systems are based on the legal duty to report certain public health information. The International Health Regulations mandate, for example, that member states of the World Health Organization (WHO) report outbreaks of plague, cholera, and yellow fever to WHO (12). Similarly, state public health departments in the United States legally mandate the reporting of cases of certain diseases from health-care providers to public health agencies (13).

Existing laws at the international and national level require reporting of a limited number of diseases, do not require systematic reporting of antimicrobial resistance, and receive inadequate compliance or noncompliance. WHO, however, has proposed including surveillance of antimicrobial resistance in the revisions of the International Health Regulations (14-16) and requiring drug resistance reporting.

In the United States, surveillance of antimicrobial resistance has been described as woeful (17). In 1992 less than $55,000 was spent on antibiotic resistance surveillance for human pathogens in the United States at the local, state, and federal levels combined (11). While the need for improved surveillance of antimicrobial resistance is recognized (1), state reporting laws have not changed much. For example, although the Council of State and Territorial Epidemiologists recommended in 1995 that states make drug-resistant *Streptococcus pneumoniae* reportable (18), not all states have (D. Bell, pers. comm.).

Several factors explain the lag of state responses to federal recommendations on resistance surveillance. First, state laws on infectious diseases often do not keep pace with new scientific findings (10). In addition, many states do not have within their legal systems the flexibility needed to respond to new threats—as was the case, for example, when the Council of State and Territorial Epidemiologists recommended that *Escherichia coli* O157:H7 be made reportable (13). Second, adding new surveillance responsibilities is difficult because of inadequate resources (13). These two factors indicate inadequate emphasis on public health within many state governments. The structure of public health law in the United States can, therefore, impede antimicrobial resistance strategies. As at the international level, if reporting is not required, surveillance for antimicrobial resistance is compromised.

Changes in diagnostic testing in the United States also raise public health and legal concerns about surveillance. Privatization of laboratory services by state legislatures may compromise national surveillance of emerging infectious diseases and investigation of outbreaks because many surveillance systems rely on data from state laboratories (13). In addition, because of economic pressures, hospitals increasingly rely on testing done in areas outside their jurisdiction; therefore, accurate information on infectious diseases in their areas may not be available. Diagnostic testing done by private laboratories may require closer federal regulation to protect the quality of needed surveillance data (D. Bell, pers. comm.).

Creation of a legal duty does not ensure the success of a policy. WHO member states have routinely ignored required outbreak reporting of plague, cholera, and yellow fever (4,19,20). Reporting of diseases within U.S. states is sometimes poor, haphazard, and unhelpful (21). Fulfillment of legal duties often hinges on sufficient resources. In many developing countries public health systems may be inadequate (22). Thus, financial and technical leadership is needed from national governments towards local authorities and from international organizations towards developing countries. In addition to legal requirements, national regulatory barriers may also hinder global surveillance. Such surveillance will require many countries to import and use equipment, software, and reagents to detect and report on antimicrobial resistance. Eliminating barriers could improve prospects for global surveillance. A precedent can be found in the proposed Convention on the Provision of Telecommunication Resources for Disaster Mitigation and Relief Operations, which obligates the parties, where possible, to lower or remove regulatory barriers for using telecommunication resources during disasters (23).

### Privacy Issues

Surveillance systems sometimes have to balance the privacy of the patient with the need for useful scientific and medical information and the need of the community to be protected from the spread of infectious diseases (9). As the HIV/AIDS epidemic has demonstrated, the privacy issue is particularly acute in sexually transmitted diseases. Because in some sexually transmitted diseases, e.g., gonorrhea, the infectious organisms are developing resistance to antibiotics, the inherent privacy concerns are compounded by surveillance-related privacy concerns. Increased surveillance for antimicrobial resistance may heighten privacy concerns with respect to other diseases, such as multidrug-resistant tuberculosis (MDRTB).

Different systems of law deal with privacy concerns differently, which could create legal problems for global surveillance of antimicrobial resistance. The differences between U.S. and European Union policies illustrate this difficulty. In the United States, the privacy of health-related information is of concern. Health information gathered by public health agencies is regulated largely by the Constitution and by state statutes. While the Constitution "requires reasonable levels of privacy and security when the government collects personally identifiable data through . . . disease reporting" (9), a recent survey of U.S. state legislation on public health information privacy concluded that many states' safeguards of public health privacy are insufficient (24). Laws protecting private sector records are even weaker (9). Some states regulate private dissemination of health-related information through physician-licensing systems, common-law tort rules on invasion of privacy, or statutes (25). Nevertheless, such legal regulations may not adequately protect privacy, as health-related information is increasingly manipulated electronically by health-care providers, health maintenance organizations, and insurance companies (9). Lack of laws adequately protecting the privacy of health-care information has led to calls for federal regulation (9,25,26), and some legislative proposals have been introduced in Congress (27).

The European Union has a strict law forbidding the processing of health information data without the written permission of the patient (28). This law places other strict conditions on the use of health data directly affecting European Union surveillance efforts. The contrast between American and European legal protection for health-related information may affect global surveillance efforts because European law permits states to withhold personal data from those who cannot adequately protect these data (26).

A particularly important development is the growth of privately owned infectious disease information. Private initiatives are building global information-sharing networks on various disease issues through the Internet and other information technologies (4,29,30); private companies are starting to monitor and test bacterial resistance globally (31); and some for-profit companies gather and sell epidemiologically useful information. These private efforts raise legal questions: privacy issues arise with the dissemination of epidemiologic data by private companies; this dissemination is treated differently in different countries; jurisdictional problems arise regarding legal regulation of information sharing in cyberspace (32) (the quality of health information on the Internet, for example, is being questioned [33-35]); and legal (and ethical) concerns arise with the practice of selling epidemiologic data, especially with data gathering, and whether governments can compel disclosure of privately gathered information in the interest of public health.

### Biological Weapons

While most of the pathogenic agents considered to be the most likely candidates for use as biological weapons do not exhibit resistance (36), the potential use of resistant pathogens as weapons is of concern because resistance blunts one of the few lines of defense against a biological weapons attack. For the U.S. Department of Defense, antibiotic resistance is one of the criteria for characterizing suspicious outbreaks of infectious disease that might point to a possible biological weapons event (37).

The main source of international law on biological weapons, the 1972 Biological Weapons Convention, prohibits the development, production, and stockpiling of biological and toxin weapons (38). Negotiations are under way to strengthen the Biological Weapons Convention through a protocol that both establishes compliance procedures and commits states to improving domestic and global surveillance of infectious diseases (37). Calls for reform are also being made in domestic legal systems (37). The comprehensive statutory and regulatory system in the United States that governs the acquisition, use, and transfer of biological agents that pose a threat to public health (39) might serve as a model for legislation in other countries.

### Personal Control Measures

The authority of public health officials to detain or isolate persons infected with highly contagious and resistant pathogens in order to protect the community is another important legal issue. MDRTB, for example, is a threat to public health because it is highly infectious (10). To deal with its TB epidemic, New York City has issued dozens of orders to detain MDRTB patients for isolation and compulsory treatment (40). Controversy exists about the proper scope of detaining patients for infectious disease control purposes (13). While U.S. courts have upheld detaining infected patients to protect public health (41), governments today face heightened judicial scrutiny of personal control measures in the public health context (10,42). In one case in New Jersey, the court held that public health authorities, in applying a 1912 TB control statute, had to comply with contemporary notions of due process and the Americans with Disabilities Act in order to detain and isolate a patient with MDRTB (43). At a time when antimicrobial resistance may have created a greater need for personal control measures for public health (e.g., with MDRTB), the status of U.S. law on the scope and nature of the government's power to undertake such measures seems unsettled (13). U.S. public health law may need to be modified to allow public health officials to control demonstrated threats of risk through flexible policy options that minimize infringements on individual rights (10).

The proper scope of personal control measures may appear pertinent only to industrialized countries, given that only those countries can identify resistant pathogens and their human hosts. In addition, the notion of personal control measures against drug-resistant malaria patients in Africa seems far-fetched, given the scale of the problem. Nevertheless, the importance of international human rights law to effective public health policies—as seen in the context of HIV/AIDS (44)—demonstrates that complacency towards individual rights in any public health policy is dangerous legally and medically.

### Rational Use of Antimicrobial Drugs

Antimicrobial drug misuse in both industrialized and developing countries is a problem in connection not only with human treatment, but also with food production (11,45,46). More rational use of antimicrobial drugs in every country—for disease treatment and food production—must be at the core of the response to antimicrobial resistance.

Education has been suggested as the primary tactic for improving antimicrobial drug use (1,16). WHO has recommended "a code of practice for prudent use of antimicrobials in food animal production" (47).

However, the global scope of antimicrobial resistance indicates that an integrated strategy operating at both the national and international legal levels is needed. Such an integrated strategy faces political and legal challenges. Building effective national legal regimes regarding prudent antimicrobial use in many developing countries is unrealistic absent financial, political, and legal support from the international community (46). International legal harmonization of principles for prudent antimicrobial drug use will have to include monitoring and enforcement, as well as financial, technical, and legal assistance by industrialized countries to developing countries.

The international legal strategy needed will be difficult to create. WHO's limited powers to adopt regulations (48) do not seem to extend to creating regulations regarding the use of drugs. WHO has not, for example, proposed revising the International Health Regulations to rationalize the use of antimicrobial drugs. WHO has authority to adopt a convention on the use of antimicrobial drugs (49), but it has not done so (4). As illustrated by its proposal for a code of practice, rather than regulations, for antimicrobial use in food animal production, WHO historically has preferred not to use international legal powers to advance global health. Lessons from international environmental efforts suggest that international law must play a major role in setting international standards for implementation domestically and creating the political, technical, and financial conditions necessary to integrate international and national law (19).

The misuse of antibiotics in food production also raises concerns under international trade law. If antimicrobial drugs routinely used by food producers lose their effectiveness against animal-borne or plant-borne diseases, exports of contaminated food may be restricted by countries applying sanitary and phytosanitary measures (SPS measures) under the World Trade Organization (WTO) and other international trade agreements to keep such food out of their territories as threats to human, animal, or plant health (50). In addition, exports might be disrupted by countries applying SPS measures under international trade agreements against products suspected of containing harmful residues of antimicrobial drugs. The Codex Alimentarius Commission already sets standards for residues from veterinary drugs in food through its Committee on Residues of Veterinary Drugs in Foods, and WHO has recommended that this Codex committee discuss antimicrobial resistance (47). Codex standards have become important in international trade law through the WTO Agreement on the Application of Sanitary and Phytosanitary Measures (SPS Agreement) (50), which uses these standards as a basis for international harmonization of SPS measures. The importance of Codex food safety standards to international trade law was seen in the Beef Hormones Case, in which the WTO held that the European Union violated the SPS Agreement for not providing scientific justification for a beef hormone regulation stricter than the relevant Codex standards (51-53).

The inability of many governments to regulate antimicrobial drug use and the consequent misuse of these drugs raise the possibility that countries might use trade restrictions on food produced with improper use of antimicrobial drugs. In this situation, the food products might have residue levels below those established as maximums by Codex, but the trade restriction is intended to pressure the exporting country to improve regulation of antimicrobial drug use and the process by which the product is made. In the context of environmental protection, trade restrictions seeking to change a production process in another country, rather than to protect against health dangers from a particular product, have been ruled incompatible with international trade law (54-56). Although “relevant processes and production methods” (SPS Agreement, art. 5.2) form part of a risk assessment under the SPS Agreement, the risk must be a specific health risk from the product (e.g., highly inconsistent residue levels created by inadequate antimicrobial regulation) rather than fear of the health consequences of antimicrobial misuse. To avoid losing trade restrictions as part of a general strategy to combat antimicrobial misuse, legitimate trade restrictions against countries that systematically neglect recognized principles and practices for antimicrobial use might be considered; such a move would elevate the status of Codex’s Code of Practice for Control of the Use of Veterinary Drugs and Guidelines for the Establishment of a Regulatory Programme for Control of Veterinary Drug Residues in Foods, as the SPS Agreement has elevated the importance of Codex’s Maximum Residue Levels for Veterinary Drugs in Foods.

The International Conference on Harmonization, a multilateral effort between the United States, Japan, and the European Union to harmonize pharmaceutical regulatory systems, is another forum for discussing antimicrobial drug resistance. If national regulatory systems begin to grapple with the overuse of antimicrobial drugs, the International Conference on Harmonization might provide a forum to discuss a harmonized approach to more rational drug use in the United States, Japan, and the European Union. As structured, the Conference does not include other countries where overuse of antimicrobial drugs is a serious problem.

National laws for improving antimicrobial drug use face difficulties in industrialized countries; realistic national strategies confront legal and political hurdles. In the United States, state legislatures probably have the power to regulate how physicians prescribe antimicrobial drugs, but any attempt to legislate more rational use of drugs might evoke negative reactions from physicians and their medical associations, who might oppose the government’s efforts to interfere with their professional judgment (57). If formal legislative regulation would not prove feasible, an alternative would be self-regulation by the medical and veterinary professions through practice guidelines, for example (57). A peer review process to monitor antimicrobial drug use has also been recommended (1). Managed care organizations may be included in the effort to control misuse of antimicrobial drugs, given their power and economic incentives to curb such misuse by physicians.

Formal regulation of antimicrobial use may be needed, which would involve monitoring and enforcement. In the United States, Congress could regulate use of antimicrobial drugs by monitoring interstate commerce in these products. Congress probably does not have the authority to regulate antimicrobial prescription practices directly; such authority rests with the states. The U.S. Food and Drug Administration (FDA) has authority to restrict the postapproval marketing of new drugs designed for treating serious or life-threatening illnesses and has indicated that these regulations can be used specifically in cases of new antimicrobial drugs (17,58). Restricted distribution is, however, a disincentive to the development of new drugs, and the regulations do not address misuse of existing products. Another regulatory strategy available to the FDA for dealing with resistance in existing products is to modify labeling requirements; modifying labels is, however, somewhat cumbersome (17). Effective regulatory changes in the United States will be jeopardized by the lack of similar changes in other countries.

Perhaps the most powerful U.S. federal strategy would be to make implementation of state policies to curb the misuse of antimicrobial drugs mandatory before states receive federal funds earmarked for public health. Although states might argue that this would encroach on their traditional public health rights and powers, such a federal enactment would be constitutional. In addition, using federal funds to improve antimicrobial use policies nationwide fits with the need for federal political leadership as well as financial and technical assistance. State governments might, therefore, welcome federal money conditioned on implementing policies the money supports. Pharmaceutical companies, worried about the federalization of policies affecting their economic relationships with local and state health-care providers, might oppose these mandates. In addition, federal leadership in this way would also run counter to trends in other areas, such as welfare reform, which are moving responsibilities from the federal to the state level.

In countries where governments subsidize the purchase of antimicrobial drugs, legislative or regulatory changes in these subsidies could lead to a decline in the use of the drugs. When Iceland ended government subsidies of antimicrobial drugs, their use in Iceland declined, while sales kept increasing in other Nordic countries (59). Although Iceland's legislative change was made for political, not medical reasons (59), it illustrates the possible impact of legislative and regulatory controls on the use of antimicrobial drugs.

Improving physicians' awareness of antimicrobial resistance does not address, however, misuse of these drugs by patients (60). MDRTB has developed largely because of improper adherence by patients to TB therapy (10); directly observed therapy was initiated as a result. Because patient misuse of antimicrobial drugs also contributes to the public health crisis, proper completion of antimicrobial therapy must also be addressed.

### New Antimicrobial Drug Research and Development (R&D)

As the antimicrobial arsenal shrinks, new drug R&D becomes critical (1). Legislation at the domestic level would ensure adequate funding for public sector involvement. For diseases that pose an especially large or complex problem (e.g., malaria), international law can play a role by structuring international cooperation through such institutions as WHO or the World Bank. The recently proposed Multilateral Initiative on Malaria, an international multiagency malaria control program, involves, for example, plans to coordinate R&D on antimalarial products supported by WHO and the World Bank (61-63).

While public involvement and funds are important, the real engine of pharmaceutical development is the private sector. However, pharmaceutical companies have only recently brought new antimicrobial drugs forward for regulatory approval. Critical disincentives continue to constrain private R&D investment in new drugs: intellectual property protection, regulatory approval procedures, and perceived antitrust law limitations on collaborative R&D.

### Intellectual Property Protection

Pharmaceutical companies fear that their R&D efforts can be undermined by loss of intellectual property rights. Lack of secure patents deters pharmaceutical companies from some R&D activities on new drugs. While the WTO Agreement on Trade-Related Aspects of Intellectual Property Rights offers pharmaceutical companies better international legal rules on patent protection (64), the loss of patented agents remains a concern. Many WTO member states, especially in developing countries, have to upgrade their national laws to fulfill the agreement obligations, a process that could take years. International law on intellectual property protection is, thus, a critical piece of the overall strategy against antimicrobial resistance.

Other patent issues are relevant to the problem of antimicrobial resistance. Pharmaceutical companies that developed antibiotics years ago but never commercially exploited them might pursue more antimicrobial R&D if their earlier antibiotics (now without patent protection) were given extra legal protection, either under patent law or a legal regime like the Orphan Drug Act (17). The efficacy of such legal strategies depends, of course, on the number of promising antibiotics potentially in the R&D pipeline.

### Regulatory Approval Procedures

Another legal deterrent to antimicrobial development is the varied, complex, and costly regulatory approval procedures pharmaceutical companies face in the United States and other countries. Unilateral efforts are being made in the United States and the European Union to streamline drug approval regimes, and the International Conference on Harmonization represents an international effort at harmonization.

Another approach involves creating "expedited approval of new antimicrobials" (1). FDA, which has already created accelerated approval rules for drugs that treat serious or life-threatening illnesses, has indicated that these rules could be used to review new antibiotic treatments (17).

### Antitrust Law Limitations on Collaborative R&D

In some circumstances, collaborative R&D efforts by a number of pharmaceutical companies might be indicated. Pharmaceutical companies have, however, noted the difficulties that antitrust laws create for collaborative R&D efforts. For example, in response to calls for collaborative R&D on antimalarial drugs, pharmaceutical companies are concerned about sharing intellectual property and about laws against cartels (65). Both U.S. antitrust law and European Union competition law, however, permit collaboration in certain areas (66,67). The Inter-Company Collaboration for AIDS Drug Development and the developing Multilateral Initiative on Malaria set precedents and might be replicated in the area of antimicrobial resistance.

### Conclusion

Strategies to address antimicrobial resistance as a public health and legal challenge must consider three levels of interdependence: among the antimicrobial drug surveillance, use, and R&D components of the public health strategy; among the levels of law—national and international; and between the public health and legal aspects of dealing with antimicrobial resistance.

Each element of the public health strategy against antimicrobial resistance affects and depends on the other elements. More rational use of antimicrobial drugs and increased R&D depend on accurate surveillance. Limitations on the use of new drugs negatively affect the incentives pharmaceutical companies have to develop new drugs. Continued misuse of antimicrobial drugs places more pressure on both surveillance and new R&D. The effectiveness of new drugs will have to be measured by accurate surveillance and will continue to be undercut by their misuse. The lack of new R&D will force continued use of existing products, reducing their effective life-span. Thus, any public health strategy addressing antimicrobial resistance must consider all three components.

Because antimicrobial resistance is a global problem, national legal reforms taken in one or a few countries would suffer if other countries did not take similar actions. For example, since drug-resistant pathogens travel easily in today's world, national legal reforms to rationalize antimicrobial use in a few countries might be subverted if such misuse is not curtailed in many other countries. The creation of new international legal duties would likewise be undermined if such duties were not translated into national law. Thus, any legal strategy against antimicrobial resistance must be pursued at both the national and international levels.

Achieving the public health objectives of an antimicrobial resistance strategy involves, at each stage, legal considerations and often calls for legal decisions. National and international law are integral to the public health mission in every country; the interdependence of public health and law forms part of the large multidisciplinary challenge created by the global problem of emerging infectious diseases.

Confronting antimicrobial resistance requires not only a scientific and public health strategy but also a legal strategy. Including law in the developing discourse will broaden and strengthen the strategy for combating antimicrobial resistance.

### Acknowledgments

I thank Dr. Polly F. Harrison, Terry P. O'Brien, Dr. David Bell, Fred Cate, Beth Cate, Roger Dworkin, Ken Dau-Schmidt, Larry Gostin, Mary Ann Torres, Cynthia Baraban, and members of the Institute of Medicine's Forum on Emerging Infections for their help and encouragement during the preparation of this article; I also thank the anonymous reviewers of Emerging Infectious Diseases for their comments and suggestions.

An earlier draft of this article was originally presented as a working paper to the Institute of Medicine's Forum on Emerging Infections in July 1997. Research for this article was supported by a research grant from the Indiana University School of Law-Bloomington.

Mr. Fidler is an associate professor of law at Indiana University School of Law-Bloomington, where he teaches public and private international law. Mr. Fidler is at work on a book entitled International Law and Infectious Diseases, to be published by Oxford University Press.

### References

1. Institute of Medicine. Emerging infections: microbial threats to health in the United States. Washington: National Academy Press, 1992.
2. National Science and Technology Council Committee on International Science, Engineering, and Technology (CISET) Working Group on Emerging and Re-Emerging Infectious Diseases. Infectious disease—a global health threat. Washington: The Committee; 1995.
3. World Health Organization. World health report 1996: fighting disease, fostering development. Geneva: The Organization; 1996.
4. Fidler DP. Return of the fourth horseman: emerging infectious diseases and international law. Minnesota Law Review 1997;81:771-868.
5. Berkley SF. AIDS in the global village: why U.S. physicians should care about HIV outside the United States. JAMA 1992;268:3368-9.
6. Gellert GA, Neumann AK, Gordon RS. The obsolescence of distinct domestic and international health sectors. J Public Health Policy 1989;10:421.
7. Fidler DP. The globalization of public health: emerging infectious diseases and international relations. Indiana Journal of Global Legal Studies 1997;5:11-51.
8. Institute of Medicine. The future of public health. Washington: National Academy Press; 1988.
9. Gostin LO. Public health law: a review. Current Issues in Public Health 1996;2:205-14.
10. Gostin LO, Burris S, Lazzarini Z, Maguire K. Improving state law to prevent and treat infectious disease. New York: Milbank Memorial Fund; 1998.
11. American Society for Microbiology. Report of the ASM task force on antibiotic resistance. Washington: The Society; 1995.
12. World Health Organization. International health regulations. Geneva: The Organization; 1983.
13. Fidler DP, Heymann DL, Ostroff SM, O'Brien TP. Law and emerging and re-emerging infectious diseases: challenges for international, national, and state law. The International Lawyer 1997;31:773-99.
14. World Health Organization. Antimicrobial resistance monitoring program. Geneva: The Organization; 1997.
15. O'Brien TF, Stelling JM. WHONET: an information system for monitoring antimicrobial resistance. Emerg Infect Dis 1996;1:66.
16. World Health Organization. Division of emerging and other communicable diseases surveillance and control, strategic plan 1996–2000. Geneva: The Organization; 1996. No. WHO/EMC/96.1.
17. Institute of Medicine. Orphans and incentives: developing technologies to address emerging infections. In: Harrison PF, Lederberg J, editors. Washington: National Academy Press; 1997.
18. Jernigan DB, Cetron MS, Breiman RF. Minimizing the impact of drug-resistant *Streptococcus pneumoniae* (DRSP). JAMA 1996;275:206-9.
19. Fidler DP. Globalization, international law, and emerging infectious diseases. Emerg Infect Dis 1996;2:77-84.
20. Fidler DP. The role of international law in the control of emerging infectious diseases. Bulletin de l'Institut Pasteur 1997;95:57-72.
21. Berkelman RL, Bryan RT, Osterholm MT, LeDuc JW, Hughes JM. Infectious disease surveillance: a crumbling foundation. Science 1994;264:368-70.
22. O'Brien E. The diplomatic implications of emerging diseases. In: Cahill K, editor. Preventive diplomacy: stopping wars before they start. New York: Basic Books and the Center for International Health and Cooperation; 1996. p. 244-68.
23. International Telecommunication Union. Working draft of the convention on the provision of telecommunication resources for disaster mitigation and relief operations. Geneva: International Telecommunication Union, 1996.
24. Gostin LO, Lazzarini Z, Neslund VS, Osterholm MT. The public health information infrastructure: a national review of the law on health information privacy. JAMA 1996;275:1921-7.
25. Schwartz PM, Reidenberg JR. Data privacy law: a study of United States data protection. Charlottesville (VA): Michie; 1996.
26. Schwartz PM. The protection of privacy in health care reform. Vanderbilt Law Review 1995;48:295-347.
27. Health information privacy. Privacy & American Business 1997;4:9-10.
28. European Union. Directive 95/46/EC of the European Parliament and of the Council on the Protection of Individuals with Regard to the Processing of Personal Data and of the Free Movement of Such Data; 1995. O.J. 95/L281.
29. Woodall J. Outbreak meets the internet: global epidemic monitoring by ProMED-mail. SIM Quarterly: The Newsletter for the Society for the Internet in Medicine 1997 Jun [cited 1997 Sep 24]. Available from: URL: http://www.cybertas.demon.co.uk/simq/issue1/papers.html.
30. SatelLife Home Page [cited 1998 Jan 6]. Available from: URL: http://www.healthnet.org.
31. Antimicrobe spy network. Science 1997;277:185.
32. Johnson DR, Post D. Law and borders—the rise of law in cyberspace. Stanford Law Review 1996;48:1367-402.
33. McKenzie BC. Quality standards for health information on the internet. SIM Quarterly: The Newsletter for the Society for the Internet in Medicine [serial online] 1997 Dec; Available from: URL: http://www.cybertas.demon.co.uk/simq/issue3/editorial.html.

34. U.S. Food and Drug Administration. FDA and the internet: advertising and promotion of medical products. 1996 Oct 17 [cited 1997 Dec 19]. Available from: URL: http://www.fda.gov/opacom/morechoices/transcript1096/fdainet9.html.
35. World Health Assembly. Cross-border advertising, promotion and sale of medical products using the internet. Geneva: The Assembly; 1997. Resolution WHA50.4, May 12, 1997.
36. Franz DR, Jahrling PB, Friedlander AM, McClain DJ, Hoover DL, Bryne WR, et al. Clinical recognition and management of patients exposed to biological warfare agents. JAMA 1997;278:399-411.
37. Kaldec RP, Zelicoff AP, Vrtis AM. Biological weapons control: prospects and implications for the future. JAMA 1997;278:351-6.
38. Convention on the prohibition of the development, production, and stockpiling of bacteriological (biological) and toxin weapons and on their destruction. Signed at Washington, D.C., London, and Moscow on Apr 10, 1972. Available from URL: http://www.acda.gov/treaties/bwc2.htm.
39. Ferguson JR. Biological weapons and U.S. law. JAMA 1997;278:357-60.
40. Ryan F. The forgotten plague: how the battle against tuberculosis was won—and lost. Boston: Little, Brown; 1993.
41. Richards EP. The jurisprudence of prevention: the right of societal self-defense against dangerous individuals. Hastings Constitutional Law Quarterly 1989;16:329-92.
42. Gostin LO. The resurgent tuberculosis epidemic in the era of AIDS: reflections on public health, law, and society. Maryland Law Review 1995; 1-131.
43. City of Newark vs. J.S., 652 A.2d 265 (N.J. 1993).
44. Gostin LO, Lazzarini Z. Human rights and public health in the AIDS pandemic. Oxford: Oxford University Press; 1997.
45. World Health Organization. Antibiotic use in food-producing animals must be curtailed to prevent increased resistance in humans. Geneva: The Organization; 1997. WHO Press Release WHO/73, 1997 Oct 20.
46. Witte W. Medical consequences of antibiotic use in agriculture. Science 1998;279:996-7.
47. World Health Organization. The medical impact of the use of antimicrobials in food animals. Geveva: The Organization, 1997. Report of a WHO meeting, 1997 Oct 13-17.
48. World Health Organization. Geneva: WHO Constitution, art. 21.
49. World Health Organization. Geneva: WHO Constitution, art. 19.
50. World Trade Organization. Agreement on the application of sanitary and phytosanitary measures. Geneva: The Organization; 1994. No. MTN/FA II-A1A-4.
51. World Trade Organization. Panel report on EC measures concerning meat and meat products (hormones). Geneva: The Organization; 1997. WTO Doc. No. WTO/DS26/R/USA, 1997 Aug 18.
52. World Trade Organization. Appellate body report on EC measures concerning meat and meat products (hormones). Geneva: The Organization; 1998. WTO Doc. Nos. WT/DS26/AB/R and WT/DS48/AB/R, 1998 Jan 16.
53. Fidler DP. Trade and health: the global spread of diseases and international trade. German Yearbook of International Law. In press 1997.
54. General Agreement on Tariffs and Trade. Dispute settlement panel report on United States restrictions on imports of tuna. International Legal Materials 1991;30:1594-623.
55. General Agreement on Tariffs and Trade. Dispute settlement panel report on United States restrictions on imports of tuna. International Legal Materials 1994;33:839-903.
56. Schoenbaum TJ. International trade and protection of the environment: the continuing search for reconciliation. American Journal of International Law 1997;91:268-313.
57. Stephenson J. Fighting infectious disease threats via research: a talk with Anthony Fauci. JAMA 1996;275:173-4.
58. Accelerated approval of new drugs for serious or life-threatening illnesses, 21 C.F.R. Sect. 314.520 (1992).
59. Stephenson J. Icelandic researchers are showing the way to bring down rates of antibiotic-resistant bacteria. JAMA 1996;275:175.
60. McCraig LF, Hughes JM. Trends in antimicrobial drug prescribing among office-based physicians in the United States. JAMA 1995;273:214-9.
61. Butler D. Time to put malaria control on the global agenda. Nature 1997;386:535-6.
62. Gallagher R. Global initiative takes shape slowly. Science 1997;277:309.
63. Mons B, Klasen E, van Kessel R, Nchinda T. Partnership between south and north crystallizes around malaria. Science 1998;279:498-9.
64. World Trade Organization. Agreement on trade-related aspects of intellectual property rights. Geneva: The Organization; 1994. No. MTN/FA II-A1C.
65. Butler D. Can industry be wooed back in the act? Nature 1997;386:540.
66. Ross SF. Principles of antitrust law. New York: The Foundation Press; 1993.
67. Rose V, editor. Bellamy & Child common market law of competition. London: Sweet & Maxwell; 1993.

## References

[PDF_00793] Berkelman R. L., Bryan R. T., Osterholm M. T., LeDuc J. W., Hughes J. M. (1994). Infectious disease surveillance: a crumbling foundation.. Science.

[PDF_00747] Berkley S. F. (1992). AIDS in the global village. Why US physicians should care about HIV outside the United States.. JAMA.

[PDF_00937] Butler D., Maurice J., O'Brien C. (1997). Can industry be wooed back into the act?. Nature.

[PDF_00927] Butler D., Maurice J., O'Brien C. (1997). Time to put malaria control on the global agenda.. Nature.

[PDF_00861] Ferguson J. R. (1997). Biological weapons and US law.. JAMA.

[PDF_00788] Fidler D. P. (1996). Globalization, international law, and emerging infectious diseases.. Emerg Infect Dis.

[PDF_00848] Franz D. R., Jahrling P. B., Friedlander A. M., McClain D. J., Hoover D. L., Bryne W. R., Pavlin J. A., Christopher G. W., Eitzen E. M. (1997). Clinical recognition and management of patients exposed to biological warfare agents.. JAMA.

[PDF_00929] Gallagher R. (1997). Global initiative takes shape slowly.. Science.

[PDF_00750] Gellert G. A., Neumann A. K., Gordon R. S. (1989). The obsolescence of distinct domestic and international health sectors.. J Public Health Policy.

[PDF_00805] Gostin L. O., Lazzarini Z., Neslund V. S., Osterholm M. T. (1996). The public health information infrastructure. A national review of the law on health information privacy.. JAMA.

[PDF_00869] Gostin Lawrence O. (1995). The resurgent tuberculosis epidemic in the era of AIDS: reflections on public health, law, and society.. MD Law Rev.

[PDF_00785] Jernigan D. B., Cetron M. S., Breiman R. F. (1996). Minimizing the impact of drug-resistant Streptococcus pneumoniae (DRSP). A strategy from the DRSP Working Group.. JAMA.

[PDF_00852] Kadlec R. P., Zelicoff A. P., Vrtis A. M. (1997). Biological weapons control. Prospects and implications for the future.. JAMA.

[PDF_00924] McCaig L. F., Hughes J. M. (1995). Trends in antimicrobial drug prescribing among office-based physicians in the United States.. JAMA.

[PDF_00931] Mons B., Klasen E., van Kessel R., Nchinda T. (1998). Partnership between south and north crystallizes around malaria.. Science.

[PDF_00774] O'Brien T. F., Stelling J. M. (1995). WHONET: an information system for monitoring antimicrobial resistance.. Emerg Infect Dis.

[PDF_00812] Schwartz Paul M. (1995). Protection of privacy in health care reform.. Vanderbilt Law Rev.

[PDF_00921] Stephenson J. (1996). Icelandic researchers are showing the way to bring down rates of antibiotic-resistant bacteria.. JAMA.

[PDF_00880] Witte W. (1998). Medical consequences of antibiotic use in agriculture.. Science.

